# Effects of Transcranial Alternating Current Stimulation on Cognitive Functions in Healthy Young and Older Adults

**DOI:** 10.1155/2016/4274127

**Published:** 2016-05-19

**Authors:** Daria Antonenko, Miriam Faxel, Ulrike Grittner, Michal Lavidor, Agnes Flöel

**Affiliations:** ^1^Department of Neurology, NeuroCure Clinical Research Center, Charité Universitätsmedizin, Charitéplatz 1, 10117 Berlin, Germany; ^2^Center for Stroke Research, Charité Universitätsmedizin, Charitéplatz 1, 10117 Berlin, Germany; ^3^Department for Biostatistics and Clinical Epidemiology, Charité Universitätsmedizin, 10117 Berlin, Germany; ^4^Department of Psychology, Bar Ilan University, 52900 Ramat Gan, Israel

## Abstract

Recently, transcranial alternating current stimulation (tACS) has emerged as a tool to enhance human cognitive processes. Here, we provide a brief summary of the rationale behind tACS-induced effects on task-relevant brain oscillations and associated cognitive functions and review previous studies in young subjects that have applied tACS in cognitive paradigms. Additionally, we present pilot data where we administered theta-tACS (6 Hz) over the temporoparietal cortex and a supraorbital reference for 20 min during implicit language learning in healthy young (mean/SD age: 22/2) and older (mean/SD age: 66/4) adults, in a sham-controlled crossover design. Linear mixed models revealed significantly increased retrieval accuracy following tACS-accompanied associative learning, after controlling for session order and learning success. These data provide the first implementation of tACS during cognitive performance in older adults and support recent studies suggesting that tACS in the theta frequency range may serve as a tool to enhance cognition, possibly through direct modulation of task-relevant brain oscillations. So far, studies have been heterogeneous in their designs, leaving a number of issues to be addressed in future research, including the setup of electrodes and optimal stimulation frequencies to be employed, as well as the interaction with age and underlying brain pathologies in specific patient populations.

## 1. Introduction

The modulation of ongoing brain activity with transcranial electrical stimulation has been shown to result in changes on the behavioral and neuronal level [1–3]. Most evidence exists for transcranial direct current stimulation (tDCS) that modulates the neuronal response threshold in a polarity-specific manner by inducing depolarization or hyperpolarization [1, 4]. This technique has been implemented most often and is currently understood most clearly [5]. Beneficial effects of tDCS have been demonstrated on the behavioral as well as neuronal level in various cognitive domains and study populations [6–10]. Only in the last few years, transcranial alternating stimulation (tACS) has attracted attention as a promising alternative approach aiming to directly interact with ongoing oscillatory cortical activity and, as a consequence, to enhance cognitive processes in healthy adults (for recent reviews, see [1, 11–13]).

In tACS, a sinusoidal current is applied to the scalp, oscillating above and below zero with a given stimulation strength (i.e., peak-to-peak amplitude) in a particular frequency (cf. [13]). Whereas tDCS modulates the excitability thresholds of neuronal membrane potentials [1, 4], tACS directly interacts with ongoing neuronal activity during cognitive or sensory-motor processes, leading to an entrainment or synchronization of brain network oscillations [1, 2, 13, 14]. Ideally, the stimulation frequency is chosen within the range of the human electroencephalography (EEG) frequency band and close to the frequency of the predominant oscillations of neuronal networks and cognitive processes under study [11–13, 15]. As brain oscillations are known to represent various brain functions, certain frequencies reflecting particular ongoing cognitive or sensory-motor processes [11, 13], tACS enhances ongoing processes through exogenous augmentation of those oscillations [11, 16]. Hence, the potential of tACS may lie not only in the frequency-specific synchronization of neuronal networks leading to a behavioral change [12] but also beyond that in the possibility of inferring causal associations between brain oscillations and cognitive processes [1, 11, 12].

The majority of experiments that have investigated the neurophysiological effects of tACS using EEG recordings consistently confirmed that tACS in a particular frequency modulates ongoing oscillations (e.g., [17–20]). For instance, alpha-tACS increased individual alpha amplitudes directly [18] and 30 min after stimulation offset [17]. tACS interferes with ongoing brain oscillations by increasing or decreasing frequency power depending on the endogenous brain state (e.g., task versus rest), predominant EEG frequency (e.g., theta versus alpha), and site (e.g., frontal versus parietal brain regions) [20, 21]. For instance, Pahor and Jaušovec observed reduced alpha power in posterior areas after theta-tACS in resting EEG while theta power in frontal areas was enhanced [20]. Three studies have examined the tACS-induced modulation of the blood oxygenation level dependent (BOLD) response using functional magnetic resonance imaging (fMRI) [22–24]. Alpha-tACS with a centrooccipital electrode (Cz-Oz) montage induced task-related BOLD signal decreases in occipital areas [22, 24]. BOLD decreases after alpha-tACS (Cz-Oz montage) were observed in (nonstimulated) temporal and frontal brain areas [22]. Cabral-Calderin et al. failed to find the expected occipital BOLD decrease after alpha-tACS (Cz-Oz montage) but rather observed overall tACS effects in regions not activated by the task [23]. In particular, the authors showed BOLD increases in frontoparietal areas during beta-tACS (16 Hz). Altogether, research findings with regard to tACS-related neuronal effects are rather scarce and complex, showing that effects depend on the task, frequency, and intensity under study and emphasize the need for further investigations.

Neurophysiological as well as behavioral tACS-induced effects have more extensively been studied in the sensory (e.g., auditory [25], visual system [19, 24, 26]) and motor domain [23, 27] (see also [2, 11] for reviews). Moreover, tACS effects on clinical symptoms due to tinnitus [28] or Parkinson's [29] and recovery after stroke [30] have been evaluated. Here, we focus on studies that examined tACS-induced modulation of cognitive functions in healthy adults, specifically executive functions and working memory capacity as well as learning and memory formation. Noteworthily, we will omit the discussion of studies that have applied slow oscillatory stimulation during sleep to promote memory consolidation (e.g., [31–33]) as it reflects a special form of oscillatory stimulation, representing a combination of direct and alternating current stimulation [11, 21]. For a combined review and discussion of sleep-oscillatory stimulation studies, see [11, 12]. The tACS interventions reviewed here assessed cognitive performance after [20, 34–36] and during [37–43] stimulation, using similar designs to those of tDCS approaches, and are summarized in Table 1. 

*Executive Control*. Six studies have examined tACS-induced enhancement of executive control processes with parietal [20, 43] and frontal stimulation targets [20, 39–41, 43, 44]. Tasks under study included problem-solving (Raven's progressive matrices) [20, 39, 44], decision-making (Balloon Analog Risk Task [40], perceptual and value-based rating [43]), and conflict processing (color-location Simon tasks) [41].

Pahor and Jaušovec recorded participants' EEG and performance on tests of fluid intelligence after theta-tACS or sham stimulation was applied [20]. The authors found increases in frontal theta and decreases in posterior alpha power followed by tACS-induced performance increases in both, the frontal and the parietal stimulation groups, with more prominent effects in the parietal group. The superior performance after tACS compared to sham in the parietal stimulation group was ascribed to the tACS-induced increase in working memory storage capacity. On the contrary, Santarnecchi and his colleagues observed increased performance speed in a similar task during gamma-tACS over frontal areas [39]. Gamma-tACS significantly improved logical reasoning abilities compared to alpha-, theta-, and beta-tACS (which all showed no effect) and sham stimulation. In a more recent study, the authors confirmed the specific gamma-tACS-induced enhancement of logical problem-solving, while spatial working memory was found to be unaffected. Further, individual differences in tACS responsiveness were supported by the observation that beneficial effects particularly emerged in participants with slower response times at baseline [44].

Sela et al. compared decision-making under risk performance during theta-tACS between left- and right-frontal and sham stimulation groups [40]. Participants in the left-frontal tACS group showed a riskier response strategy compared to right-frontal and sham stimulation groups which was interpreted as a disruption of risk-averse decision-making due to tACS-induced interhemispheric imbalance. The authors failed to find the expected enhancement of decision-making strategies during right-frontal tACS. Similarly, Polanía et al. observed more inaccurate choices between food rewards during tACS which induced oscillatory frontoparietal desynchronization [43]. In a recent study investigating tACS effects on conflict processing, van Driel et al. observed reduced response times on low-conflict trials (resulting in reduced response conflict) during midfrontal theta-tACS compared to an alpha-tACS control [41]. The authors concluded that theta-tACS leads to a more cautious (i.e., slower) response strategy. 

*Memory*. Six studies have examined tACS-induced enhancement of working memory [34–38, 42] while one study looked at declarative memory [45]. As for executive functions, stimulation targets in all studies either were frontal [34–38, 42, 45] or also included parietal sites [35, 36, 38, 42]. Tasks included memory storage and matching (letter discrimination, visual-array comparison, and *n*-back) [34–38, 42], memory span (digits and Corsi blocks) [36, 42], and word-pair learning [45].

Polanía et al.'s study was the first to look at frontoparietal tACS during a visual memory-matching task. Participants were faster during phase synchronized (0°) and slower during phase desynchronized (180°) theta-tACS compared to sham stimulation [38]. These findings were interpreted as causal evidence that theta phase coupling between frontal and parietal brain areas is crucial for cognitive performance (cf. [11]). A further study confirmed the beneficial effects of theta-tACS on verbal working memory accuracy compared to sham stimulation with a bifrontal electrode setup [37]. The authors also found an association between tACS-induced improvements and subsequent retrospective self-evaluation.

Jaušovec and his colleagues found higher memory scores after parietal theta-tACS compared to sham and no effects of frontal stimulation [35, 36]. In addition, the authors demonstrated a latency decrease of the event-related potential component P300 during task performance indicative of an improved capability to solve the task after tACS [35]. Vosskuhl and his colleagues could not replicate the beneficial tACS effects on backward memory span and memory matching (i.e., *n*-back task) in a frontoparietal electrode setup along the central line using a frequency below the individual theta [42]. Apart from that, participants in the tACS group performed better in forward digit span during stimulation, reflecting short-term memory, compared to the sham group. Higher task-related theta amplitudes after stimulation in the tACS but not sham group, as assessed by EEG, confirmed successful modulation of brain oscillations. However, this study also observed that behavioral effects disappeared after tACS offset. The most recent study by Hoy and her colleagues was the first to directly compare frontal gamma-tACS with tDCS on change in working memory performance [34]. While tDCS had unexpectedly no effects compared to sham, tACS significantly enhanced performance at higher memory loads (i.e., 3-back task) compared to sham and tDCS. Noteworthily, the authors observed considerable baseline differences between stimulation conditions that impede their conclusions.

The first study that looked at tACS-induced effects on declarative memory (excluding the sleep and slow oscillatory stimulation studies, see above) applied tACS in the hippocampal ripple range (140 Hz) during the encoding of word pairs and found that while a night of sleep induced forgetting (i.e., reduction of the number of recalled word pairs) after sham, there was no such difference between immediate (after stimulation) and delayed (after sleep) recall after tACS [45]. tACS and sham condition did not per se differ in immediate or delayed recall.

*In sum*, recent studies on cognitive processes in healthy young samples indicate successful modulation of brain oscillations and behavioral outcome through frontal or parietal stimulation. The majority of the studies suggest a particularly beneficial effect of tACS in the theta frequency (either fixed for all study participants or determined individually). Oscillations in the theta frequency are associated with memory processes and reflect the encoding of new information [46]. Thus, they mirror cortical communication with the hippocampus and support cognitive operations such as learning [47]. While executive and working memory processes have been examined in several studies, the investigation of tACS-induced improvement of learning processes is underrepresented. Although the studies point to effectiveness of tACS, there is substantial heterogeneity in the study designs and various stimulation parameters that restrict overall conclusions. Moreover, previous studies only included healthy young subjects. Given that task-related as well as resting brain oscillatory activity may show altered patterns across the human lifespan [48–51], the impact of stimulation protocols on older healthy adults, and even more so on older adults with emerging brain pathology, is particularly relevant but so far unknown.

*Our Study*. In our randomized, sham-controlled pilot study, we asked whether retrieval of implicitly learned associations between objects and pseudowords can be improved by concurrent theta-tACS over the left temporoparietal cortex. We used the same paradigm and electrode montage as Flöel et al. who showed that accuracy in retrieval after learning was improved by anodal tDCS compared to sham and cathodal stimulation [52]. Here, we examined the effects of temporoparietal theta-tACS (6 Hz) on performance accuracy. To the best of our knowledge, no previous study has explored tACS effects on implicit learning success so far. Moreover, this study is the first administering tACS in a group of older adults.

## 2. Materials and Methods

### 2.1. Participants and Study Design

Twenty-four healthy young and older adults participated in the study (see Table 2 for participant characteristics). All were native German speakers, were right-handed, and had no history of neurological or psychiatric disorders. All older participants underwent neuropsychological testing prior to study inclusion in order to assure normal cognitive functioning (CERAD-Plus, https://www.memoryclinic.ch/de/). Performance levels on all cognitive domains lay within age- and education-related norms. The study was approved by the ethics committee of the Charité University Medicine and conducted in accordance with the Helsinki Declaration. Written informed consent was obtained from all participants prior to participation.

**Figure 1:**
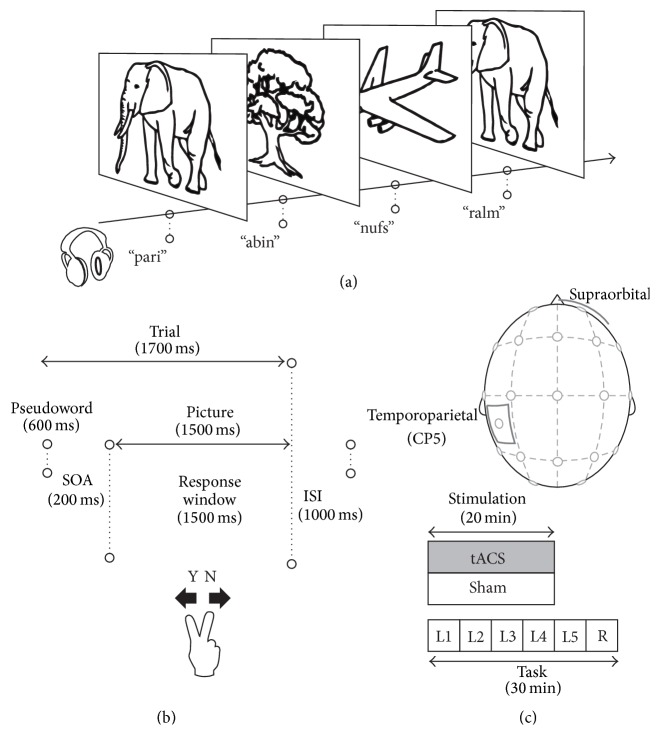
(a) Sample auditory (presented via headphones) and visual (presented on the screen) stimuli of the experiment. (b) Duration and composition of each trial (120 in total in each block). (c) Illustration of the electrode positions and experimental design. The two stimulation electrodes were placed over the left temporoparietal area (CP5; 5 × 7 cm^2^) and the right supraorbital area (10 × 10 cm^2^). L1–L5, learning blocks 1–5. R, retrieval testing.

We implemented a single-blind sham-controlled crossover design. Participants underwent two stimulation conditions (tACS and sham) in a counterbalanced order. Sessions were separated by seven days to avoid carryover effects and were administered at the same time of the day. During stimulation, participants were given a language learning paradigm [52, 53]. Two older participants were excluded from further analysis due to difficulties in understanding task instructions, as indicated by subjects' pressing only one of the two response buttons throughout the entire task.  Figure 1 illustrates the task and study design.

### 2.2. Transcranial Alternating Current Stimulation

A battery-driven stimulator (NeuroConn, Ilmenau, Germany) was used to deliver alternating current to the scalp via two sponge-electrodes soaked into saline solution. One electrode (5 × 7 cm) was placed centrally over the CP5 area (left hemisphere) according to the 10-10 EEG system; the other (10 × 10 cm) was placed over the right supraorbital area (see Figure 1(c) for electrode position). This electrode montage has been used in previous studies investigating tDCS-induced effects in the same [52] and an explicit vocabulary learning task [54]. Electrodes were fixed with two rubber bands and impedance was kept below 5 kOhm. Stimulation parameters were set as follows: 6 Hz, 20 min (7200 cycles), 10 s fade in/out, 0° phase, and 1 mA peak-to-peak amplitude. Placebo or sham stimulation, respectively, was delivered identically to previous studies [52, 54] where current was applied only for 30 sec.

### 2.3. Language Learning Paradigm

The paradigm was adapted from previous studies (see [52, 53, 55] for detailed description) and presented using the software Presentation (Neurobehavioral Systems, http://www.neurobs.com/, version 18.1). In brief, the paradigm consists of the presentation of pseudoword-picture pairs. For each participant, a set of 30 pseudowords and 30 visually presented pictures of daily life objects were randomly matched up to 30 “correct” pairings. Two different sets of stimuli existed for the two experimental sessions (cf. [52]).

During the learning phase of the experimental session, five training blocks with 120 trials each were presented (600 trials in total). “Correct” pairings (e.g., /pari/ and elephant) occurred ten times (twice per block); in addition, each picture occurred ten times with varying “incorrect” pseudowords (e.g., /ralm/ and elephant) (see Figure 1(a) for sample stimuli). “Incorrect” pairings were shown only once. The order of trial presentation was randomized. Response buttons were reversed for one-half of the participants. In each trial, the picture was presented 200 ms after the onset of the auditory spoken pseudoword (all normalized to the same loudness and a length of 600 ms). During the picture presentation, participants had to decide whether the pairing was “correct” or “incorrect” by button press. This time window was raised up to 1500 ms compared to 1000 ms in [52, 53, 55] in order to adapt the experiment to older adults (see Figure 1(b) for trial timings).

During the subsequent retrieval phase, learning success was measured in a “transfer” block. Here, instead of visually presented objects, corresponding spoken German words were presented together with the pseudowords with identical stimuli count, trial timings, and underlying frequency principle. Completion of the experimental session took 35 min in total; hence, stimulation was administered during the first four learning blocks (see Figure 1(c) for study design). Both accuracy and reaction times were assessed during each block. Main outcome measure was the accuracy on the retrieval block.

### 2.4. Questionnaires

Before and after stimulation mood ratings were administered using the Positive and Negative Affect Schedule (PANAS) [56]. Participants rated their positive and negative affect (10 items each) on a scale ranging from 1 to 5, where higher values describe more positive or negative feelings, respectively. After completion of the second experimental session, participants were asked to retrospectively report the occurrence of potential adverse effects (symptoms like tingling, itching, burning, and headache) during stimulation in a standardized questionnaire as well as blinding effectiveness [57] (“tACS first session”; “tACS second session”; “do not know”).

### 2.5. Statistical Analysis

Statistical analyses were conducted with IBM SPSS 23 (http://www-01.ibm.com/software/de/analytics/spss/). Linear mixed models (random intercept models) [58] were calculated for task performance (retrieval and learning) as well as for the mood ratings (differences in positive and negative affect) with repeated measurements as level-one units nested in individuals who were level-two units. Models included the factors condition (tACS and sham) and group (old and young) as well as their interaction in order to examine differences of stimulation effects between groups. For the main outcome variable retrieval performance, the model was adjusted for mean learning performance (derived from the learning phase) to control for overall task-related performance and session effects. We additionally included the interaction between mean performance and condition in order to test whether tACS effects were dependent on initial performance levels. The regression coefficient for this interaction was not significant (*β* = −.17, SE = .22, *F*_(1,29)_ = .59, and *p* = .45) and was therefore excluded in the final model. For the learning variable, linear and quadratic terms for time points (centered) were entered into the model in order to describe the learning curve throughout the blocks. The model was adjusted for session order effects and included a group by learning curve interaction in order to test for differences in learning curves between groups. Model-based post hoc pairwise comparisons of the estimated fixed effects were computed. A two-sided significance level of *α* = 0.05 was used. No adjustment for multiple testing was applied.

## 3. Results

### 3.1. Retrieval Performance

**Figure 2:**
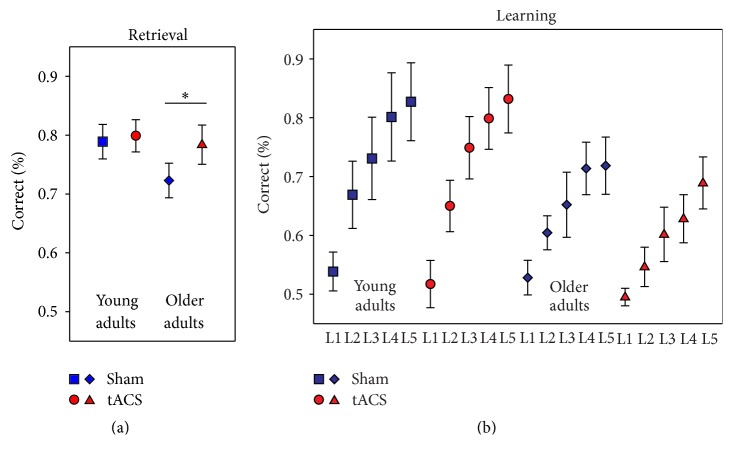
(a) Retrieval performance in the transfer task which directly followed the learning blocks. Model-based estimates are depicted. *N* = 22 subjects/44 measures. ^*∗*^ *p* < .05. (b) Learning performance in the five learning blocks. Blue rectangles/rhombs for sham condition in young/older adults, red circles/triangles for tACS condition in young/older adults. *N* = 22 subjects/220 measures. Means and 95% CI.

Figure 2(a) depicts model-based means for retrieval performance separately for condition and age groups. The linear mixed model revealed a significant effect of condition, indicating superior performance in the tACS compared to sham condition (mean difference (95% CI): .04 (.01–.07), *F*_(1,18)_ = 6.36, and *p* = .021). A significant effect of group further indicated superior performance in young compared to older adults (mean difference (95% CI): .04 (.003–.08), *F*_(1,21)_ = 5.13, and *p* = .034). Interaction of condition and age group showed a trend towards significance (*β* = .05, SE = .03, *F*_(1,18)_ = 3.25, and *p* = .089). Model-based post hoc tests showed a significant difference between conditions in the older (mean difference (95% CI): .06 (.02–.11); *p* = .010) but not the young (mean difference (95% CI): .01 (−.03–.05); *p* = .586) group.

### 3.2. Learning Performance

Task performance improved over the learning blocks in both groups (Figure 2(b)). Improvement was significant with regard to the five learning blocks in a curvilinear convex manner indicated by a significant linear increase (*β* for the five blocks (centered and linear) = .07, SE = .003, *F*_(1,173)_ = 625.18, and *p* < .001) and an additional significant coefficient for the square of block order (*β* for the five blocks (squared) = −.01, SE = .002, *F*_(1,173)_ = 28.66, and *p* < .001). A significant interaction of group and block indicated flatter learning curves in older adults (*β* = −.03, SE = .005, *F*_(1,173)_ = 29.87, and *p* < .001). Interaction of age group and condition was not significant (*β* = −.04, SE = .04, *F*_(1,39)_ = .86, and *p* = .36). Model-based post hoc tests did not show significant differences in learning between the tACS and sham condition neither in older (mean difference (95% CI): −.04 (−.10–.02); *p* = .171) nor in young (mean difference (95% CI): −.004 (−.06–.05); *p* = .873) adults.

### 3.3. Mood Ratings and Stimulation Side Effects

Mood ratings before and after stimulation are displayed in Table 3. Mixed effects models showed that there was no significant difference in mood rating changes neither on the positive nor on the negative scale between conditions (positive affect: *β* = −.14, SE = .10, and *p* = .19; negative affect: *β* = .00, SE = .05, and *p* = 1.0) or groups (positive affect: *β* = −.07, SE = .11, and *p* = .528; negative affect: *β* = .05, SE = .05, and *p* = .280).

Both groups tolerated the stimulation well in both conditions. Only few participants reported adverse effects.  Table 4 shows the amount of participants retrospectively reporting the occurrence of the respective adverse events for the conditions. Note that none of the participants reported the occurrence of phosphene perception or visual sensations. In line with typical sensations induced by tDCS [59], participants could not differentiate between tACS and sham stimulation (young adults: four incorrect and three correct guesses and five “do not know”; older adults: one correct guess, four “both sessions,” and seven “do not know”).

## 4. Discussion

The current pilot study explored the effects of theta-tACS over the left temporoparietal cortex with a right supraorbital reference during implicit language learning in young and older adults, in a sham-controlled randomized crossover design. The results suggest that tACS application during implicit learning improved subsequent retrieval performance, after controlling for learning and session effects. This effect was driven by the superior performance of older adults in the tACS compared to the sham condition. Effects of tACS on learning performance and mood ratings were not significant.

### 4.1. tACS Effects on Cognitive Functions

Our results of a tACS-induced performance enhancement are in line with other studies that have applied tACS in the cognitive domain (and are reviewed above, Table 1). Consistently, research studies that have administered theta-tACS over the posterior (parietal) brain area with a contralateral reference electrode observed stimulation-enhanced after-effects on working memory capacity and executive functions [20, 35, 36]. These studies of the Jaušovec group compared frontal and parietal tACS effects between groups finding more pronounced behavioral improvement in the parietal stimulation group [20] for tests of fluid intelligence (i.e., problem-solving and visual-spatial reasoning) and no effect in the frontal stimulation group [35, 36] on memory storage capacity. The authors hypothesized that their findings may indicate a specific memory storage enhancement through parietal theta-tACS versus attention enhancement through frontal theta-tACS. In contrast to our study as well as all other studies reviewed above, stimulation in the studies of the Jaušovec group has been applied before the task under study instead of during task performance.

Two studies have applied frontal high-frequency tACS during word-pair encoding [45] and working memory processing [34] showing tACS-induced behavioral benefits after stimulation. Equivalent to our results, no effects emerged during stimulation. While Hoy et al. observed selective performance improvement, Ambrus et al. showed reduced forgetting after a night of sleep. In contrast to our study, Ambrus et al. applied tACS bifrontally in the ripple range (140 Hz) with mastoid reference electrodes in order to use the electrode setup of sleep studies [31, 32]. Moreover, in the present study, an implicit associative learning paradigm was used which mimics naturalistic language acquisition with high ecological validity [53, 55], rather than explicit word-pair encoding [45]. Implicit learning paradigms may be particularly suitable for stimulation-accompanied training interventions in older adults [60]. Altogether, these observations provide further support for the potential of tACS to produce sustained after-effects [17, 18]. However, the exact underlying mechanisms remain unknown. We speculatively interpret the fact that the observed offline effect on retrieval found in our study may reflect the impact of parietal theta oscillations during associative learning on learning-induced plasticity, without altering the actual learning curve.

Other research studies in the cognitive domain have demonstrated online tACS effects in working memory [37, 38, 42], short-term memory [42], and cognitive control tasks [39–41, 44]. Two of these studies have also assessed performance after stimulation which was unaffected [37, 42]. None of the previous studies have applied tACS during a learning process where paired associations have to be encoded (except [45]). In sum, due to their different paradigms, designs, and stimulation parameters, results cannot be easily compared between studies.

This is the first study to use theta-tACS during associative learning in healthy participants and the first to implement tACS in older adults. Previous studies have provided evidence for tACS effects in young adults. Moreover, the most recent study by Santarnecchi and his colleagues suggests that individual baseline performance levels may account for tACS responsiveness [44], similar to what has been shown for tDCS-induced effects particularly in older adults [61]. However, due to age-related brain network alterations [49, 51], it is unknown whether beneficial effects and underlying mechanisms transfer to the aged population. Research studies on tDCS-induced cognitive improvement and neuronal enhancement suggest that stimulation effects may differ between young and older adults with potentially divergent neuronal mechanisms [3, 7]. We provide the first evidence for the feasibility of tACS in older adults.

### 4.2. Limitations

Several limitations have to be considered when interpreting the results of our study. First, the sample size was rather modest. However, it was comparable to similar previous studies (see Table 1), and we chose a statistical model that included the whole sample of young and older adults. Second, only stimulation in the theta frequency was applied based on theoretical considerations ascribing a central role of theta in cognitive and particularly memory processes [16, 46, 47] and on previous tACS studies using this frequency to improve memory processes [35, 36, 38]. This theta frequency was set to 6 Hz with no individual variation. Note though that previous studies have also found beneficial effects with fixed stimulation frequencies [20, 35, 36, 41, 42], suggesting that individual variation may not necessarily be required [21]. However, as no control conditions were applied, we cannot conclude that the effects were specific to the electrode montage or frequency applied. Third, stimulation setup resembled tDCS studies, as did most other tACS investigations. To date, it is not clear whether observations from tDCS studies including the optimal (and maximal) stimulation duration, intensity, or electrode montages can be translated to tACS at all [13].

### 4.3. Outlook

The heterogeneity of study designs (within versus between subjects), tasks domains (executive functions, working memory, and declarative memory), electrode montages (frontal and parietal site of active electrode and number and position of electrodes), stimulation parameters (frequency and oscillatory phase, intensity, duration, and electrode sizes), and control conditions (sham stimulation and different frequencies) hampers comparisons between studies, as well as joint conclusions so far [11, 13]. Further, conclusive interpretation needs combined evidence from both recoding (i.e., EEG or fMRI) and stimulation techniques (cf. [39]). However, approaches including both behavioral and brain physiological outcomes before, during, and after stimulation are difficult to realize from a technical point of view and still scarce [11]. Only few studies assessed tACS effects on both cognition and brain function [20, 35, 42]. Moreover, the possibility of analyzing physiological effects during tACS is still limited (EEG: [19], fMRI: [23, 24]). In the cognitive domain, only one study assessed behavioral performance before, during, and after stimulation and EEG frequency spectra before and afterwards [42].

Further, a recent technical guide by Woods and colleagues emphasizes the role of the various parameters chosen in transcranial electrical stimulation techniques [62]. Particularly, tACS compared to tDCS may be a more complex technique which needs additional careful consideration of stimulation parameters. The authors stress that, contrary to tDCS where one electrode serves as the active and the other as the reference electrode, in tACS, all cortical areas under the electrodes are modulated similarly. Thus, stimulation modes that are specific to tACS such as the induction of corticocortical desynchronization versus synchronization (achieved by the placement of a third electrode) still need to be refined [62]. An important approach to investigate and compare different conditions of interregional phase coupling has been presented by Polanía and his colleagues [38, 43]. We argue that tACS might be ideally suited to alter oscillations within large-scale neuronal networks that are affected by aging [50, 51].

Notwithstanding, findings of positive effects have been observed despite differences in stimulation protocols, tasks, and study populations. Hence, current empirical findings point to the potential benefit of tACS. Yet, this rather newly emerged research field still poses a number of open issues to be scrutinized in further investigations.

## 5. Conclusions

The few research studies that have investigated tACS-induced effects on higher-order cognitive performance in healthy young adults to date suggest a performance-enhancing effect of theta-tACS or gamma-tACS over frontal and parietal areas. However, study designs are heterogeneous with partly contradictory results, underlining the complexity of parameter choice for tACS (as compared to tDCS, e.g.). In our pilot study, we present the first application of theta-tACS during associative learning in healthy older adults and demonstrate a small but significant improvement in retrieval performance. These data, together with several previous reports in the field, encourage further exploration of tACS with the aim to improve cognition. However, we acknowledge that a number of questions need to be addressed now, including methodological and technical decisions about the number, position of electrodes, stimulation frequency, duration, and timing [62], before tACS should be introduced as a means to ameliorate age-related cognitive decline.

## Figures and Tables

**Table 1:** Summary of studies that have applied tACS to enhance cognitive functions in healthy adults, in alphabetical order.

Authors	Participants and design	Domain, task, and DV	Electrode montage	Stimulation parameters	Main results	Conclusion
Ambrus et al. 2015 [45]	*N* = 18 (age 25 ± 3) Crossover design (tACS versus sham)	Memory/declarative memory Associative word-pair learning test Accuracy (cued recall) after encoding and after a night of sleep	L DLPFC (F3) + L mastoid/R DLPFC (F4) + R mastoid	*Frequency*: 140 Hz *Intensity*: 1 mA peak-to-peak amplitude *Duration*: 10 min *Electrode size*: 5 × 5 cm^2^ (F3/4) and 5 × 7 cm^2^ (mastoids) *Timing*: task (encoding)	Forgetting (accuracy decrease) after a night of sleep in sham but not tACS condition *#Offline-effects*	Potential effect of 140 Hz-tACS on consolidation

Hoy et al. 2015 [34]	*N* = 18 (age 29 ± 7.7) Crossover design (tACS versus tDCS versus sham)	Executive function/working memory 2- and 3-back task before and after stimulation Change in *d*-primes and accurate RT	F3 + R supraorbital	*Frequency*: 40 Hz (tACS); NA (tDCS) *Intensity*: 1.5 mA peak-to-peak amplitude (tACS); 2 mA (tDCS) *Duration*: 20 min *Electrode size*: both 5 × 7 cm^2^ *Timing*: task (2-back)	Selective improvement on 3-back after gamma-tACS compared to tDCS and sham No effects of RT *#Offline-effects*	Role of frontal gamma-tACS in neuromodulation Preliminarily evidence for gamma-tACS preferentially improving working memory performance at higher loads

N. Jaušovec and K. Jaušovec 2014 [35]	*N* = 12 in P3 and *N* = 12 in F3 tACS group (age 20 ± .4) Crossover within-group design (tACS versus sham)	Attention/working memory Visual-array comparison task EEG ERP amplitudes and latencies and memory capacity score	P3 (L) or F3 (L) + R supraorbital (Fp2)	*Frequency*: 5 ± 1 Hz (based on individual alpha peak minus 5 Hz) *Intensity*: 1–2.2 mA (based on individual skin sensations) *Duration*: 15 min *Electrode size*: both 5 × 7 cm^2^ *Timing*: rest	Increased capacity score after P3-tACS and no effect after F3-tACS Parietooccipital P300 ERP component latency decrease *#Offline-effects*	Causal relation between working memory storage capacity and theta frequency oscillations in the left parietal area

Jaušovec et al. 2014 [36]	*N* = 12 in P3 and *N* = 12 in P4 and *N* = 12 in F3 tACS group (age 20 ± .3) Crossover within-group design (tACS versus sham)	Executive function/working memory Corsi-block tapping, digit span (both forward and backward), and *n*-back Span core and number of correct responses	P3 (L), P4 (R), or F3 (L) + R supraorbital (Fp2)	*Frequency*: 5 ± 1 Hz (based on individual alpha peak minus 5 Hz) *Intensity*: 1–2.2 mA (based on individual skin sensations) *Duration*: 15 min *Electrode size*: both 5 × 7 cm^2^ *Timing*: rest	Increased capacity score after L and R parietal tACS, but not frontal, compared to sham More pronounced effect of L parietal tACS on backward compared to forward recall *#Offline-effects*	Central role of parietal areas for working memory storage capacity

Meiron and Lavidor 2014 [37]	*N* = 12 in tACS and *N* = 12 in sham (age 19–27) Between-subjects design	Executive function/working memory Verbal working memory task (2-back task with word pairs) and rating of success-confidence on task Accuracy and self-evaluation scores	L DLPFC (F3/AF3 midpoint) + R DLPFC (F4/AF4 midpoint)	*Frequency*: 4.5 Hz *Intensity*: .5 mA peak-to-peak amplitude *Duration*: 20 min *Electrode size*: 4 × 4 cm^2^ *Timing*: task (verbal *n*-back)	Improved online accuracy in bilateral tACS compared to sham WM enhancement related to self-evaluation scores *#Online-effects*	Increased functional connection between working and retrospective monitoring through tACS

Pahor and Jaušovec 2014 [20]	*N* = 14 in parietal and *N* = 14 in frontal tACS group (age 21 ± .3) Crossover within-group design (tACS versus sham)	Executive function/cognitive control/fluid intelligence (problem-solving and visual-spatial reasoning) Progressive matrices, paper folding, and cutting test EEG frequency spectra and performance test scores	L parietal (P3) or L frontal (F3) + R supraorbital (Fp2)	*Frequency*: 5 ± 1 Hz (based on individual alpha peak minus 5 Hz) *Intensity*: 1–2.2 mA (based on individual skin sensations) *Duration*: 15 min *Electrode size*: 5 × 7 cm^2^ (P3/F3) and 7 × 10 cm^2^ (Fp2) *Timing*: rest	Increased resting EEG theta power and decreased EEG alpha power after theta-tACS Improved test scores after tACS Behavioral effect more pronounced in L parietal tACS group (better in both tests) compared to L frontal *#Offline-effects*	Influence of brain oscillatory activity by theta-tACS Importance of parietal regions for fluid intelligence Parietal tACS increased working memory storage capacity and frontal tACS influenced attentional components

Polanía et al. 2012 [38]	*N* = 18 and *N* = 18 in control experiment (age 22–30) Crossover design (tACS versus sham)	Executive function/working memory Delayed letter discrimination task RT and correct responses	L frontal (F3) + L parietal (P3) Cz (reference electrode)	*Frequency*: 6 Hz (35 Hz in control experiment) *Intensity*: 1 mA peak-to-peak amplitude *Duration*: 14 min *Electrode size*: all 5 × 5 cm^2^ *Timing*: task (letter discrimination)	Reduced RT with synchronized 6 Hz-tACS Increased RT with desynchronized 6 Hz-tACS *#Online-effects*	Causality of frontoparietal theta phase coupling Suitability of tACS to induce coupling and decoupling of behaviorally relevant brain rhythms

Polanía et al. 2015 [43]	*N = 86* (age 20–30) Crossover design (alternating intervals of stimulation/no stimulation)	Executive function/decision-making Perceptual- and value-based decisions RT and accuracy	mPFC (Fpz) + parietal (Pz) R *s*houlder (reference electrode)	*Frequency*: 55 Hz (modulated by a 6 Hz envelope) *Intensity*: 2 mA peak-to-peak amplitude *Duration*: ~18 min *Electrode size*: 5 × 7 cm^2^ (Fpz/Pz) and 10 × 10 cm^2^ (shoulder) *Timing*: task (decision-making)	More inaccurate value-based decisions with desynchronized tACS No effect on perceptual decisions *#Online-effects*	Causal influence of degree of rhythmic synchronization between frontoparietal brain areas on value-based decision-making

Santarnecchi et al. 2013 [39]	*N* = 20 (age 21 ± 12) Crossover design (5 conditions: sham, theta-, alpha-, beta-, and gamma-tACS)	Executive function/cognitive control/fluid intelligence (problem-solving and visual-spatial reasoning) Progressive matrices RT and accuracy	L MFG (−34, 16, 30) + Cz	*Frequency*: 5, 10, 20, or 40 Hz *Intensity*: .75 mA peak-to-peak amplitude *Duration*: delivered for duration of task performance *Electrode size*: 5 × 7 cm^2^ *Timing*: task (visuospatial reasoning)	Shortening of RT in gamma-tACS condition Selective enhancement of more complex trials *#Online-effects*	Causal involvement of gamma band synchronization in higher-order cognition

Santarnecchi et al. 2016 [44]	*N* = 24 (age 23.8 ± 3.1) in Exp. 1 and *N* = 34 (age 24.3 ± 2.8) in Exp. 2 Crossover within-group design (3 conditions in Exp. 1: sham, theta-, and gamma-tACS; 3 conditions in Exp. 2: sham, tRNS, and gamma)	Executive function/cognitive control/fluid intelligence and working memory Abstract-reasoning task (progressive matrices), delayed-match-to-sample (change-localization) task, and control task (odd-even numbers) RT and accuracy	L MFG (−34, 16, and 30) + Cz	*Frequency*: 5, 40 Hz (Exp. 1); 5, 101–640 Hz tRNS (Exp. 2) *Intensity*: .75 mA peak-to-peak amplitude *Duration*: 30 min *Electrode size*: 5 × 5 cm^2^ *Timing*: task (visuospatial reasoning and working memory)	Shortening of RT to solve complex logic problem in gamma-tACS condition compared to all other conditions in both Exp. 1 and Exp. 2 Greater tACS-induced enhancement for slower participants No effect in theta-tACS or tRNS No effect on working memory task *#Online-effects*	Frequency-specific neuromodulatory effects on cognitive ability Individual differences in gamma-tACS responsiveness

Sela et al. 2012 [40]	*N* = 9 in L, *N* = 8 in R, and *N* = 10 in sham Between-subjects design (3 groups: L, R, and sham)	Executive function/cognitive control/decision-making Balloon Analog Risk Task Number of pumps and total number of balloon explosions	L DLPFC (F3) + L temporal (CP5)/R DLPFC (F4) + R temporal (CP6)	*Frequency*: 6.5 Hz *Intensity*: 1 mA peak-to-peak amplitude *Duration*: 15 min *Electrode size*: all 5 × 5 cm^2^ *Timing*: task (decision-making)	Riskier decision-making in L-tACS group compared to R-tACS and sham group No difference between sham and R-tACS groups *#Online-effects*	Theta-band oscillatory and DLPFC activity critical for decision-making Disruption of ability to process and adjust actions based on negative feedback and errors by theta-tACS

van Driel et al. 2015 [41]	*N* = 31 (age 19–31) Crossover design (theta- versus alpha-tACS)	Executive function/cognitive control/conflict processing Spatial response conflict task (color-location Simon task) RT and accuracy	Centered between FCz/Cz bilaterally on cheeks (reference electrodes)	*Frequency*: 8–12 versus 4–8 Hz (based on individual alpha and theta peak) *Intensity*: 2 mA peak-to-peak amplitude *Duration*: ~20 min *Electrode size*: 9 cm^2^ (FCz/Cz) and 35 cm^2^ (cheeks) *Timing*: task (spatial conflict)	Slower RTs and lower accuracy for high conflict trials: conflict reduced during tACS Effect driven by slower RTs in low-conflict trials *#Online-effects*	Modulation of cognitive control through theta-tACS

Vosskuhl et al. 2015 [42]	*N* = 17 in tACS and *N* = 16 in sham group (age 26 ± 3) Between-subjects design	Executive function/working memory (WM) and short-term memory (STM) Digit span backward and 3-back task (both WM) and digit span forward (STM) EEG frequency spectra, mean list length (digit span task), and accuracy (3-back task)	FPz + Pz	*Frequency*: ~3.7–4.6 Hz (below individual theta frequency) *Intensity*: ~.4–1.3 mA (below individual phosphene or perception threshold) *Duration*: 18 min *Electrode size*: all 5 × 7 cm^2^ *Timing*: task (digit span)	Increased digit span forward during stimulation in tACS group No effect in digit span backward and 3-back accuracy No effect beyond the stimulation period Increase in theta-amplitude after stimulation in tACS group *#Online- + offline-effects*	Successful manipulation of theta frequencies caused increase in STM capacity

EEG, electroencephalography; ERP, event-related potential; DLPFC, dorsolateral prefrontal cortex; DV, dependent variable; L, left; MFG, middle frontal gyrus; mPFC, medial prefrontal cortex; R, right; RT, reaction times; tACS, transcranial alternating current stimulation; tDCS, transcranial direct current stimulation.

**Table 2:** Participant characteristics.

	Young adults	Older adults
Age mean ± SD (years)	22.3 ± 1.5	66.3 ± 3.9
*N* (f)	12 (6)	12 (6)
Education mean ± SD (years)	15.5 ± 1.4	15.8 ± 3.2

**Table 3:** Mean and standard deviations of positive and negative mood ratings.

		tACS		Sham	
		Pre	Post	Pre	Post
Young adults	PA	2.8 ± .6	2.8 ± .6	2.8 ± .7	3.0 ± .6
	NA	1.2 ± .2	1.1 ± .1	1.1 ± .1	1.1 ± .2

Older adults	PA	3.5 ± .8	3.4 ± .9	3.3 ± .7	3.3 ± .9
	NA	1.1 ± .3	1.1 ± .4	1.2 ± .4	1.1 ± .3

PA, positive affect; NA, negative affect.

**Table 4:** Number of participants reporting adverse events during stimulation.

		tACS	Sham	Both
Young adults	Pain	—	1	1
	Tingling	—	3	6
	Itching	4	1	—
	Burning	—	2	2
	Tiredness	—	4	—
	Loss of concentration	1	1	1

Older adults	Tingling	—	—	3
	Itching	—	1	1
	Tiredness	1	2	1
	Loss of concentration	—	4	2
	Headache	—	2	—

Adverse events that were not reported by any of the participants are not listed. *N* in each group = 12.
